# Making malaria control a priority: a lesson for today's malaria community

**DOI:** 10.5281/zenodo.14718884

**Published:** 2025-01-22

**Authors:** Anton Alexander

**Affiliations:** 1 BC Business Centrum, Elscot House, Arcadia Avenue, London N3 2JU, United Kingdom.

## Abstract

For malaria control to be successful, experience has shown that success is more likely where all involved feel the attempt must not be allowed to fail, and that success can be the only acceptable outcome. Importantly, all those at the top must have such commitment, and, in particular, this should also include the funder, the source of finance of the attempt. That would be malaria control treated as a priority. If not treated as such, experience has shown the outcome of such attempts to be disappointing.

This paper will briefly study three long-forgotten but successful projects, each with different objectives and ultimate goals. Each objective made it necessary to treat malaria control as a first critical preliminary step before setting out to achieve each different principal goal. Failure to control malaria would have made each ultimate goal impossible. In other words, the objective of malaria control had to be a priority if the principal goal was ever to be achieved. The paper will then proceed to contrast these three accounts with today's attempts at malaria control and elimination. I begin with a reminder of basics for the reader.

*Malaria control* is the reduction of disease incidence, prevalence, morbidity or mortality to a locally acceptable level as a result of deliberate efforts. Continued interventions are required to sustain control.

*Malaria elimination* is the interruption of local transmission (reduction to zero incidence of indigenous cases) of a specified malaria parasite in a defined geographical area as a result of deliberate activities. Continued measures to prevent re-establishment of transmission are required [1].

Since the end of the nineteenth century, malaria has been known as a local problem because mosquitoes travel only a limited distance from their breeding sites to bite and feed on humans. If that mosquito got infected during a prior bloodmeal, when it bites, it will inject malaria parasites, thereby transmitting the disease. Therefore, destruction of mosquito breeding sites can prevent or control malaria in that locality.

The paper will deal principally with malaria control, and only very briefly with malaria elimination. To eliminate malaria in any locality, it is first necessary to control the disease in that locality. Malaria elimination can usually then only be achieved through continuous and sustained malaria control, by ensuring those breeding sites remain unproductive for mosquitoes for longer periods of time.

The three successful projects that are reported here all took place in Palestine approximately 100 years ago. Each project should be considered separately in its own right, but with certain basic malaria control principles applying to all three. Because of the extent and severity of the disease, also because of the varied backgrounds of the inhabitants (there were very few inhabitants initially at that time), Palestine was to serve as an ideal classroom for instruction in malaria control. In 1919, Dr. Manson-Bahr, a future director of the London School of Hygiene and Tropical Medicine, had described Palestine as one of the most highly malarious countries in the world [2]. The severity of the disease had desolated the whole country, reducing it in many areas to being either uninhabitable or almost empty of inhabitants.

The disease had decimated the population to the point that Mark Twain in 1867 wrote of his visit to Palestine:

*"A desolation is here that not even imagination can grace with the pomp of life and action...We never saw a human being on the whole route." [3]*

In its 1876 Handbook for Palestine and Syria, the travel agent Thomas Cook and Son said of Palestine that:

*"Above all other countries in the world, it is now a land of ruins. In Judea it is hardly an exaggeration to say that...for miles and miles there is no appearance of present life or habitation, except the occasional goatherd on the hillside, or gathering of women at the wells, there is hardly a hill-top of the many within sight which is not covered with the vestiges of some fortress or city of former ages." [4]*

To emphasise this, the opening line of the League of Nations Malaria Commission main report after having inspected the anti-malaria works then in progress in Palestine in 1925 began:

*"[Palestine] is a small country and, as a whole, thinly populated..." [5]*

The successful malaria-control of 100 years ago thereafter in fact enabled the area to become one of the most densely populated regions in the world today.

Firstly, anyone wishing to initiate successful malaria control should view any attempt at control as not being allowed to fail. History has shown those intending successful malaria control needed such an outcome as a priority from the outset. Perhaps, upon reflection, it was because of the severity of the disease in Palestine at that time that tackling it, anyway, obviously had to be a priority.

There are theoretically different strategies that can be adopted to achieve malaria control, some more practical, more proven and more likely of success than others, but whatever strategy or approach is chosen, malaria control must remain a priority. A practical reason for treating successful malaria control to be the only acceptable outcome is that whatever strategy is chosen, more attention is likely to be given to any anti-malaria task which, therefore, will more likely be carried out thoroughly, not just partially but completely. There can be no cutting corners. Partial or incomplete anti-malaria work is usually pointless and a waste of time and resources. In this respect, this paper will later contrast the thoroughness involved in the three Palestine examples with the thoroughness encountered with many current malaria management attempts elsewhere in the world that have often disappointingly appeared to either struggle or have been at best temporarily effective.

Experience has also shown that if malaria control is important enough, if it is truly a priority, a leadership will find a way, a practical strategy or method to succeed. Uncomfortably, and embarrassingly for many readers of this paper, if it is not a priority, experience has also shown a leadership will instead find an excuse (and place the blame for a failure elsewhere).

## Three examples of successful malaria control

The paper will now briefly summarise the three successful malaria control examples, which reveals that each of these was in fact a by-product of a 'main project' where the severity of the malaria, if left uncontrolled, would have frustrated or blocked the ultimate goal of each 'main project'.

The three 'main projects' took place or commenced in Palestine and were:

1. The defeat of the Turkish Army by the British Army in 1918;
2. Commencing in 1922, making a Jewish Homeland in Palestine;
3. Archaeological excavations by the eminent archaeologist Sir Flinders Petrie in 1930.

If malaria had not been controlled, 1) the Turkish Army might have been left in control of Palestine after 1918, 2) it would be unlikely that Israel could ever have come into existence, and 3) a major archaeological excavation might not have taken place.

Successful malaria control was in fact a consequence, a necessary by-product, of these projects where malaria had to be confronted before the final goals of the main projects could be achieved. Undefeated malaria would have been an overwhelming obstacle that would have denied a successful outcome for each project. Therefore, managing malaria had to become a priority. Unless control of malaria had first been achieved, or at least commenced, the goal of dealing with the projects would have been practically impossible. Dealing first with malaria was to become an obvious priority.

### 1. The defeat of the Turkish Army by the British Army in 1918.

The Palestine battlefront was the only one of all the malarious battlefronts in all the theatres of war during WWI where the disease was successfully controlled [6]. By December 1917, the British Army, under the command of General Allenby, had advanced northwards from Egypt against the Turkish army and had occupied the southern half of Palestine. However, finding itself in such a malarious area, consideration was given by the British Army to withdrawing south to a healthier position to keep the troops safe, but it was decided instead to remain where it was and to deal with the disease as a priority. Accordingly, once the decision to remain had been taken, from April to September 1918, the British Army spent almost six months in the area it occupied thoroughly destroying the breeding sites of the anopheline mosquito, and ensuring these breeding sites remained free of larvae. Such destruction of the breeding sites was conducted under the direction of an experienced entomologist, Major E. Austen, using two thousand of its own troops and, importantly, also a great number of labourers of the Egyptian Labour Corps (ELC). Allenby thereby effectively protected his army from malaria whilst it retrained and regrouped for the final and decisive battle against the Turkish Army.

The reader must appreciate the severity of malaria in Palestine at that time, including the danger the disease represented there. It will also explain the advantage that Allenby gained due to the anti-malaria work which had kept the army healthy for when battle was to commence in September. In a published paper by one of Allenby's senior medical officers, it was stated that had no anti-malaria work been undertaken by Allenby, his army would have 'simply melted away' [6]. Allenby was later to comment after his victory that he had previously been informed the incubation period for malaria was 7–10 days after anyone was bitten by an infected mosquito. He commented that he had taken this incubation period into account when planning his attack. In fact, the British Army was to decisively defeat the Turkish Army within the 10-day incubation period timeframe from the 19^th^ September when Allenby's army first crossed the front line, from the 'healthy' British Army area into the Turkish positions. Allenby's calculations were proven to be correct because, approximately 10 days after the initial advance, from 1^st^ October onwards, over 20,000 British troops, over half Allenby's army began to collapse or ultimately die from malaria due to exposure to the bite of an infected mosquito beyond the British Army's 'healthy' line. But by then, however, Allenby's decisive victory over the Turkish Army had already been achieved.

### 2. Commencing in 1922, making a Jewish Homeland in Palestine

Modern Political Zionism, the movement for Jewish self-determination in Palestine, arose in the late 19^th^ century as a reaction to anti-Semitic and exclusionary nationalist movements in Europe. Anti-Jewish pogroms in Russia (1881–1884) stimulated growth of Zionism, resulting in formation of pioneering organisations and the first major wave of Jewish immigration to Palestine. Between 1882 and 1914, approximately 75,000 eastern European Jewish idealists arrived to settle in Palestine, but by 1914, about half this number had died or had left, unable to cope with the severe malarial conditions [7].

After 19^th^ September 1918, when Allenby began his cavalry charge, the anti-malaria work directed by Austen had ceased. Anopheline mosquitoes accordingly had returned and were again abundant in many formerly 'healthy' areas in Palestine. Palestine reverted to its former 'pre-Allenby/Austen' state, rendering much of the country again either uninhabitable or sparsely populated.

The Mandate for Palestine was a League of Nations mandate of the territories of Palestine and Transjordan, both of which had been conceded by the Ottoman Empire following WWI. After the 1918 defeat of the Turkish Army by the British Army, the Palestine Mandate, on behalf of the League of Nations, was assigned to the United Kingdom, and operated from 1920 to 1948 by a British civil administration.

At the conclusion of WWI, sustainable rural malaria management by a civil authority in Palestine had been viewed by the British governing authorities as financially impossible, because it would have required considerable manpower for many years. Jewish immigrants seeking to settle in Palestine realised they had to try to undertake steps to control malaria or they would perish. Malaria control thereupon became a priority.

In 1920, Dr. Israel Kligler, an American public health scientist, and an idealistic Zionist Jew, arrived to settle in Palestine, and during 1921–1922, he began what essentially was an integrated malaria vector management campaign that was to become the first start anywhere in the world of a successful national malaria elimination campaign.

Kligler wrote of the difficulty of understanding at first sight how a country as sparsely settled as Palestine could have such a disease so widely spread and epidemics so continuous. He wrote that epidemics are usually correlated with crowding. He continued that the answer was furnished by a study of the socio-economic conditions prevailing there, and that because the country was small and undeveloped, there was a constant active movement of the various population groups including the Bedouin and those on annual pilgrimages. He noted that this movement was as effective in spreading malaria and maintaining its endemicity as it would be in the case of any other infectious disease.

Kligler set out to make malaria control durable and pointed out that education was as important as the anti-malaria work itself because unless the population at risk appreciated the ongoing need for vigilance and maintenance as directed by entomologists, malaria would return and the original destruction of mosquito breeding sites would be of little value. Without the mosquito, there can be no malaria, it was reasoned correctly.

Through his education of all the inhabitants (albeit initially there were very few at that time), Kligler was able to rely on Arab and Jewish voluntary co-operation of entire communities—men, women, and children—to assist in the anti-malaria work. This co-operation remained very strong throughout, despite attempts to break it by those few extremist Arab elements (e.g. forerunners of today's Hamas) who wished to see such cooperation fail. It was important to Kligler that everyone felt involved. The cooperation endured, malaria 'control' became malaria 'elimination' and the World Health Organization eventually declared malaria eliminated in Israel in 1967.

### 3. Archaeological excavations by the eminent archaeologist Sir Flinders Petrie in 1930

Sir Flinders Petrie of the British School of Archaeology in Egypt wished to explore Tell el Ajjul, which was on the bank of the Wady Ghuzzeh, north of the border between Palestine and Egypt. The British School's 1931 literature described the site as the largest ancient Tell in Palestine. Due to the severity of malaria in the area, James Starkey, a colleague of Sir Flinders Petrie, and many of the workers at the site, were unable to continue with the dig and were confined to hospital with malignant malaria. Work was declared impossible at the site owing to the disease, and the site was closed in December 1930 by the Mandate's Department of Antiquities in Jerusalem.

Petrie was passionate to excavate the ancient site. An official Health inspector came and advised Petrie's party to consider:

*"... the digging of a canal to keep the stagnant water in the wady flowing; Petrie set his men and some prisoners from the local jail, to work and at a cost of £100 the canal was completed; it had to be cleared afresh every season."* [11]

The canal that was dug was reportedly two and a half miles in length and all nearby pools were filled up. In 1931, after several months of work, the excavation was permitted to continue. Without having first controlled the malaria in the locality, the excavations would not have been possible. Malaria control had been a priority.

## Funding malaria control

It may also be helpful to consider the funding for these projects, which colours the importance various parties attached to dealing with the disease.

### 1. The defeat of the Turkish Army by the British Army in 1918.

The funding for malaria control by the British Army was treated as a necessary incidental military expense of the campaign. Sustainable malaria control in peace-time Palestine was viewed by the British governing authorities as impossible, and a subsequent 1918 British Army Medical Authority report noted that:

*"It is interesting to speculate on what can be the future of a country such as [Palestine] from the health point of view. One cannot conceive the [malaria] problem [in Palestine] which faced the Army last spring [in 1918 during WWI] being undertaken by a Civil Authority. The expense alone would be prohibitive... The great bulk of the work [carried out by the British Army] was washed out by the first rains of October (1918)."*

The fact that the anti-malaria work was not maintained by the British after its victory, and which lack of maintenance permitted malaria to return, demonstrated malaria management had served its purpose and had abruptly ceased to be a priority after 19^th^ September. If any attempts immediately after to maintain the control would have taken place, it would have lacked funding and would have accordingly failed.

### 2. Commencing in 1922, making a Jewish Homeland in Palestine

An American lawyer, Louis B. Brandeis, had become a prominent figure in the Zionist movement, becoming active in 1912 in the Federation of American Zionists, eventually becoming the leader and spokesperson of American Zionism.

With the close of WWI, Brandeis visited Palestine in 1919, and he was greatly impressed by the seriousness of the malaria situation. Brandeis suffered from malaria, and his boyhood experience with malaria in Kentucky, USA, had left a deep impression on him. And this, coupled with his practical sense made him grasp the significance of the problem the new Zionist settlers faced. He realised that before all else, the land had to be made safe for settlement, and dealing with malaria had therefore to be a priority. It was written:

*"But a practical question, suggested by the bloated appearance of hundreds of little children, distressed him.*

*The land was filled with malaria, and he knew malaria and its evil influence from his Kentucky boyhood, when the bowl of quinine pills was always on the table. To put an end to malaria was therefore the first task he would assign to the American Zionists."*

Brandeis's commitment to Zionism was so strong that in 1921, he personally financed Kligler to conduct several significant demonstrations that involved systematic studies of the local aspects of the malaria elimination rural problem in Palestine.

The results of these demonstrations were so encouraging that the American Jewish Joint Distribution Committee (JDC), the leading global Jewish humanitarian organisation, stepped in to assume financial responsibility for the furtherance of malaria elimination in the country.

As a result, the Malaria Research Unit (MRU) was established by Kligler in Palestine and funded by the JDC for the special purpose of studying ways and means of controlling malaria, for putting the results of these investigations into practice, particularly in the rural areas affecting Jewish settlements in Palestine either present or proposed.

The establishment of the MRU relieved the Palestine Mandate Government Health Department of a part of the task which should rightly have been performed by it. The problem of malaria in Palestine was acute, but it was then never a priority for the Health Department. In any event, the Health Department did not have the means for conducting an intensive study of and campaign against malaria in the rural community as a whole. Therefore, with a view to assisting the Jewish rural population and also Arab communities (if any) in the neighbourhood, as well as relieving the government of the financial responsibility of the work it ought really to have taken on, the JDC placed at the disposal of the Government Health Department a complete unit/organisation for the study of malaria in Palestine and methods of control. Sufficient funds were provided to carry on this task for a period of a little over four years, and with reducing contributions each year thereafter [9].

The necessity for the rural anti-malaria work to have been financed mainly by Zionist charitable funds for the first years was an obvious demonstration of a lack of priority on the Mandate's part in dealing with malaria. Eventually, after the first four years, the Mandate Government had noted the success and benefits of malaria control and negotiated with the JDC to gradually share the expense and after several years, the Government then assumed full responsibility.

### 3. Archaeological excavations by the eminent archaeologist Sir Flinders Petrie in 1930

Raising funds for any archaeological dig was always very difficult and the thought of raising additional funds for anti-malaria work probably would have deterred others from taking the matter further. Petrie appealed to the Mandate Health Department for help in defraying the cost and expense of digging the canal. It appears the Department did not respond with any financial assistance at all. Petrie and his wife were obliged to raise funds through exhibitions of their archaeological finds in order to pay for the work and also for hospital bills for their workmen, presumably from hospitalisation related to the malaria.

Quite obviously, malaria control in those rural settings of the excavations was then of little importance to the Mandate authorities. Petrie could rely only on his own efforts, but his passion and enthusiasm ensured the funds were raised and malaria control was successful, subsequently permitting the archaeological excavations to continue.

## Malaria control today

Failure to control malaria would have brought about a failure of the Palestine projects. Such a possibility ensured the anti-malaria works were carried out thoroughly. The works were not carried out only partially but thoroughly, completely, and is a prime reason underpinning their success. That appears to be a significant difference between then and now.

All the examples of successful malaria management in Palestine 100 years ago had been conducted as a priority. However, many of today's attempts at malaria control will admit to a lack of thoroughness – certainly by the standard required in Palestine 100 years ago. In the eyes of those involved with malaria management in Palestine 100 years ago, many of today's efforts elsewhere would probably have appeared as merely dabbling in control of the disease!

Today's attempts at malaria elimination appear to jump straight into dealing with inhabitants and their use of bednets, bypassing the basic practical problems of initially controlling the disease. It is important the reader clearly understands why present methods are not producing the anti-malaria results that were achieved by the methods employed in Palestine 100 years ago. Importantly, there is no hint in the following of anything being conducted thoroughly - if the disease is not initially controlled in the first place, elimination of the disease will be extremely unlikely.

The following is an extract from a previous paper [10]:

I thought it would be helpful for the reader to see an illustration of current approaches to malaria elimination, and below is an example of one such approach. I would wish in particular to draw the attention of the reader to the subsequent evaluations at the end of the example, the evaluations suggesting there is something missing from such an initiative. The attempt appears to be trying to create a habit of a correct nightly use of bednets through awareness of the disease, but the evaluation below also explains why such 'awareness' alone is insufficient.

The USAID Communications and Malaria Initiatives in Tanzania (COMMIT) [12] as a behaviour change communication program was implemented between 2008 and 2012 that incorporated elements of the Theory of Planned Behaviour. The literature states it sought to increase perceptions that bednets are the socially accepted approach for avoiding malaria, foster peoples' confidence in their ability to use bednets every night and improve the attitude that malaria is an unavoidable and constant presence in people's lives. The programme sought to engage communities and individuals with common sets of messages and information through a variety of mutually reinforcing sources, including mass media, rural community outreach, and community-initiated approaches.

The programme's initial evaluation demonstrated that exposure to the activities improved the self-efficacy necessary to take action to prevent malaria. Nearly 77% of those exposed to the programme put all their children under bednets the previous night, as opposed to 34.6% of those unexposed. Exposure to the campaign significantly increased the perception that nets are effective in stopping malaria and the belief that nets are useful and easy to use.

The subsequent COMMIT Project Performance Evaluation of the impact of the programme commented on the increase in bednet use and suggested that of course awareness of the disease was heightened:

*“... , communication operates by indirect as well as direct effect. Especially when a behaviour starts becoming the norm, those not directly exposed can still be influenced by what they see and hear around them; direct exposure is not needed in order to be affected by the campaign.”*

*“Common sense, consistent testimony from people on the ground ... suggest that the millions of print materials, thousands of radio spots and community-level events, ...have had a significant impact on the malaria behaviours of many Tanzanians.”*

But the evaluation also commented that elimination of malaria demands more than just putting children under a bednet the previous night. It demands a long-term commitment. To eliminate malaria, the task of such elimination must be **viewed as a priority**, and it is doubtful the Tanzanians would have come to view the task as such merely as a result of this programme. The Executive Summary of the evaluation concluded with the following comment:

*“...it is generally accepted that Behaviour Change Communication is an essential, but not sufficient, component of any public health initiative. It is not enough to provide commodities and services: one also has to educate the public about their use and importance and build a continuing demand for them.”*

And the Project Performance Evaluation interestingly concluded with a following recommendation:

*“Awareness levels on key malaria messages are now very high. People understand the dangers of malaria and are familiar with the steps needed to prevent or treat it; however, action needs to be focused on converting that increased awareness into actual behaviour change. A more strategic phase of behaviour change work needs to occur that looks at each desired behaviour, particularly those that have been resistant to change, to better understand the barriers to achieving high levels of compliance with malaria recommendations.”*

Today, there seems to be little thought, if any, given to dealing thoroughly with the disease in the first place. It would appear that methods involved in malaria management are not seen today as a priority either by the funder at the top or by the inhabitant at the bottom. And for this reason, malaria is likely to remain a threat. Absence of resolute determinism to end malaria merely results in evolution catching up with us time and again through drug and insecticide resistance with devastating consequences.

There are expressions that speak of a perpetuation of existing routine activities - ‘business as usual’ – yet these expressions can also at the same time imply the utter pointlessness and stupidity of such continuation. Well known examples of such expressions - ‘But the band played on’ or ‘Rearranging the deckchairs on the Titanic’, - are sometimes used to imply the pointlessness of an activity when referring to the fate of the Titanic in 1912 as the ship was sinking after striking an iceberg. Dr. Pedro Alonso, former Director of the WHO Global Malaria Programme, commented:

*“Taken together, we are facing a malaria crisis. We need to do things differently. As Einstein famously said, doing the same thing over and over again and expecting a different result is insanity.”* [13]

Dr Alonso’s comment is, of course, correct.

## Conclusions

It will be noted there were ‘main projects’ in Palestine 100 years ago with their respective final objectives, and with malaria control almost as a by-product.

Sadly, simple good health today on its own appears to generally be insufficient to stir today’s politicians and policymakers into viewing malaria control as a true priority. The prevailing attitude appears to be one of fatalism, namely that malaria is inevitable and a death from malaria is merely a fact of life, whereas in fact a death from malaria today should be considered an unnecessary tragedy.

The malaria-community will often speak of its ever expanding 'toolbox' in its fight against the disease, but the community unfortunately often views the task of malaria management as only some mechanical process, a general blanket method of control or elimination of the disease. The toolbox can be useful but such a toolbox on its own is incomplete if success is to be the outcome.

As a suggestion, it possibly would be helpful if the huge economic cost of malaria in lost productivity (billions of dollars each year) could be highlighted and repeatedly emphasised by each affected government. The gains of investing in malaria control are abundantly clear and yield return on investments not matched in other economic undertakings. If the cost was perhaps not presented as a bland single figure, it may instead perhaps be helpful if it was broken down so that all inhabitants can identify certain aspects or areas with which they can relate. Perhaps each inhabitant can then appreciate what he or she is missing because of malaria's dead hand on economic growth.

Perhaps then, the prospect of a vibrant modern economy may make malaria control, and ultimately its elimination, a priority by making such an economic prospect a final objective.

## References

[1] World Health Organization. WHO Malaria Terminology (update 2021). Geneva World Health Organization. https://tinyurl.com/53f45bup (accessed 25 January 2025).

[2] Austen EE Anti-mosquito measures in Palestine during the campaigns of 1917–1918. Trans. R. Soc. Trop. Med. Hyg. 1919 13 47 62 Doi: 10.1016/S0035-9203(19)80006-8

[3] Twain M The Innocents Abroad. 1869 https://tinyurl.com/2x463yx8 (accessed 25 January 2025).

[4] Cook T and Son. 1876. Cook's Tourists' Handbook for Palestine and Syria. Simpkin Marshall & Co. London. https://tinyurl.com/37zt2tcv (accessed 25 January 2025).

[5] League of Nations: Health Organisation. Malaria Commission. Reports on the Tour of Investigation in Palestine in 1925 League of Nations, Geneva, Sept. 1925. [C.H. (Canton Helvetica) 52.]

[6] Alexander A How malaria was 'weaponised' by the British Army during World War I. MalariaWorld J. 2023 14 2 Doi: 10.5281/zenodo.8203655 PMC10395414 37538369

[7] Dunkel FV Alexander A Three stepping stones leading to malaria elimination, changing world maps on the way. MalariaWorld J. 2020 11 4 PMCID: 8415057 PMC8415057 34532223

[8] Archive of the Department of Antiquities of Mandatory Palestine (1919 – 1948). https://tinyurl.com/5tptvjkc (accessed 25 January 2025).

[9] Kligler IJ Letter dated 31^st^ December 1925 to Flexner, and agreement of 7^th^ January 1926 between Colonel Heron and Dr Kligler. Central Zionist Archives Jerusalem A612/36

[10] Alexander A Did malaria elimination begin to lose its way in 1925? "If you think education is expensive, try ignorance". MalariaWorld J. 2022 13 6 PMCID: 9838089 PMC9838089 36655234

[11] Drower MS Flinders Petrie: A Life in Archaeology. University of Wisconsin Press, Madison, 1995.

[12] USAID/Tanzania: COMMIT – Project Performance Evaluation. https://tinyurl.com/mptysmv9 (accessed 20 January 2025).

[13] Alonso P Letter to malaria partners. https://tinyurl.com/yshd9xby (accessed 20 January 2025).

